# CD22: A Regulator of Innate and Adaptive B Cell Responses and Autoimmunity

**DOI:** 10.3389/fimmu.2018.02235

**Published:** 2018-09-28

**Authors:** Edward A. Clark, Natalia V. Giltiay

**Affiliations:** ^1^Department of Immunology, University of Washington, Seattle, WA, United States; ^2^Division of Rheumatology, Department of Medicine, University of Washington, Seattle, WA, United States

**Keywords:** CD22, B cells, autoimmunity, antigens, TLR7, T cell dependent, T cell independent

## Abstract

CD22 (Siglec 2) is a receptor predominantly restricted to B cells. It was initially characterized over 30 years ago and named “CD22” in 1984 at the 2nd International workshop in Boston (1). Several excellent reviews have detailed CD22 functions, CD22-regulated signaling pathways and B cell subsets regulated by CD22 or Siglec G (2–4). This review is an attempt to highlight recent and possibly forgotten findings. We also describe the role of CD22 in autoimmunity and the great potential for CD22-based immunotherapeutics for the treatment of autoimmune diseases such as systemic lupus erythematosus (SLE).

## Introduction

CD22 is classified as an “inhibitory receptor” because it contains four ITIMs within its cytoplasmic tail. Yet to classify it simply as a receptor that inhibits B cell functions would mean ignoring data that reveal a more nuanced story. For instance, besides the two distal ITIMs in the cytoplasmic tail of CD22 that recruit the protein tyrosine phosphatase (PTP), SHP-1, another motif, Y828 (or mouse Y807), when tyrosine phosphorylated, binds Grb2 and Shc and, forms a complex with SHIP and activation of a MAP kinase pathway that can regulate cell survival and proliferation (5–7). Just how and when these two CD22 cytoplasmic domains are utilized are still not well understood. One possibility is that B cell responses to T cell independent (TI) antigens (Ags) may utilize one or both binding domains, while other receptor responses use a different domain. In support of this model, Fujimoto et al. (8) reported that BCR ligation leads to rapid tyrosine phosphorylation of both the classic ITIMs and the Grb2 recruitment motif, while CD40 ligation only induces tyrosine phosphorylation of the ITIM domains.

Within the group of B cell-associated surface molecules, CD22 stands out not only because it can physically associate with the B cell receptor (BCR), but also because crosslinking the BCR on CD22-deficient B cells induces elevated responses such as mobilization of intracellular calcium (9–11). Hence, it has been emphasized that CD22's main function is to inhibit BCR signaling (2). Yet several initial studies of CD22-deficient mice showed that CD22 regulates TLR signaling and the survival of B cells and not just BCR signaling (see below). In our initial study, we reported that CD22 KO B cells proliferated less well than WT B cells after anti-IgM treatment but better after treatment with LPS (10).

The extracellular domain of CD22 binds to α 2,6-linked sialic acid ligands linked to galactose, which are expressed on a number of cell types including hematopoetic cells, certain endothelial cells and T and B cells. The enzyme α 2,6 sialyltransferase 1 (ST6Gal1) synthesizes this ligand, and ST6Gal1^−/−^ mice phenocopy many but not all of the defects seen in CD22^−/−^ mice (12, 13). CD22 itself expresses its ligand as does surface IgM (sIgM) and CD45, so CD22 can associate with itself or other cell surface molecules on B cells in a “cis” configuration or with ligands on other cells in a “trans” configuration. Endogenous CD22-CD22 *cis*-interactions can “mask” CD22, limiting its ability for binding to ligands in *trans*. Not all CD22 expresses its ligand, so CD22 also is found on B cells in a ligand-free, “un-masked” form. The relative roles of “masked” and “unmasked” forms of CD22 working in “cis” or “trans” are presented in detail elsewhere (3, 13–15).

## Regulation of bcr signaling by CD22

The model that others and we helped to develop is as follows: After BCR ligation the protein tyrosine kinase (PTK) Lyn is activated to phosphorylate two distal ITIM motifs of CD22, which in effect then recruit the PTP, SHP-1, to come to the plasma membrane and get tyrosine-phosphorylated and activated (2–4, 13–15). Both genetic and biochemical data support the importance of this pathway. Mice with a combination of half doses of Lyn, CD22 and SHP-1 have a defective phenotype found in homozygous parents (16). Recruitment of SHP-1 (PTP.1C) to the plasma membrane may increase its enzymatic activity more than a 1,000 fold (17). Thus, there is no question that the SHP-1 associating with CD22 is ready and able to dephosphorylate its substrates.

Just what all those substrates might be in B cells still is not entirely clear. Yes, phosphorylated ITIMs can be dephosphorylated by SHP-1 *in vitro* (18, 19), but *in vitro* data do not necessarily reflect *in vivo* substrates. A phosphopeptide of the cytoplasmic tail of CD22 is not a particularly good substrate for SHP-1, unlike phosphopeptides from some other ITIM-containing receptors (20). Using a catalytically inactive trapping mutant of SHP-1, the Hozumi group showed that after BCR ligation both myosin and CD72 are substrates for SHP-1 (21, 22). SLP-76 and BLNK may also be SHP-1 substrates in B cells (23, 24).

Several studies have emphasized functions of CD22 that do not rely entirely on SHP-1. Chen et al. (25) found that CD22 can associate with plasma membrane calcium ATPase (PMCA) to enhance calcium efflux after BCR ligation; this association only occurs if CD22 is tyrosine phosphorylated. The non-ITIM Y828 site in CD22 that associates with Grb2 must be tyrosine phosphorylated for PMCA to interact with CD22, and Grb2 is required for this association (26). Chen et al. (25, 26) propose that PMCA regulates Ca^2+^ in B cells through its interaction with CD22 via a SHP-1-independent pathway. Grb2 has been previously implicated in the negative regulation of Ca2+ in B cells through its localization by the adaptor protein Dok-3 to the plasma membrane and subsequent inhibition of Btk (27). CD22, which like Dok-3 is a substrate for Lyn, may help to facilitate this process.

Most studies examining the role of CD22 in BCR signaling have used biochemical assays. Han et al. in a different approach used *in situ* photoaffnity crosslinking of glycan ligands to CD22 (28). Their results showed recognition of formation glycans of neighboring CD22 molecules, forming homomultimeric complexes, suggesting that CD22 is distributed in membrane microdomains, which the authors suggested restricts CD22 interactions with other glycoproteins. More recently, Gasparrini et al. (29) used super-resolution microscopy to examine the interactions of CD22 with the actin cytoskeleton. They found that CD22 works within the “cortical cytoskeleton” to regulate BCR signaling including tonic signaling and that it is organized into nanodomains. Simple inhibition of actin polymerization with latrunculin A led to rapid tyrosine phosphorylation of both CD22 and SHP-1. Using advanced microscopic methods such as dual-color structured illumination microscopy, they found that IgM, IgD, CD19, and CD22 exist on the cell surface of resting B cells in “preformed but distinct islands,” with some co-localization. CD22 was not randomly distributed but rather more likely to be found in clusters about 100 nm in radius. *In silico* modeling showed that a high lateral mobility of CD22 nanoclusters would enable CD22 to come in contact with many BCR nanoclusters and thereby regulate tonic or Ag-induced signaling. Indeed, CD22, when tracked, turned out to be highly mobile, able to diffuse about four to five times faster than either sIgD or CD19 and nearly twice as fast as sIgM. The authors suggested that this would enable CD22 to mediate “global BCR surveillance.”

Interestingly, Gasparrini et al. (29) also found that the extent of CD22 nanoclustering is regulated by the PTP, CD45; the less CD45 on B cells, the larger the CD22 nanoclusters were and the slower CD22 diffused. CD45 expresses α-2,6 sialic acid and, like CD22, is a CD22 ligand (30, 31). A reduction or absence of CD45 most likely leads to more CD22-CD22 homotypic interactions and thus larger clusters. Couglin et al. (32) also implicated extracellular CD45 in the regulation of CD22. They found that expression of transgenes encoding either extracellular CD45 without its cytoplasmic domain or CD45 with a catalytically inactive form of CD45 in CD45^−/−^ mice rescued B cell defects seen in these mice such as elevated basal Ca^2+^ levels but not T cell defects. This effect required CD22.

Recently, the crystal structure of the first three extracellular domains (ECD) of human CD22 was deduced at a 2.1 A resolution (33). Strands of domain 1 elongate and extend into a ß-hairpin that shapes a preformed binding site for the sialic acid ligand. Analysis of CD22 molecules including a full length CD22 ECD revealed that CD22 is relatively inflexible and behaves as a tilted “elongated rod,” which does not change its conformation much after ligand binding (33). The authors propose that “the elongated, tilted CD22 structure—and the location of its binding site at the N-terminus—is ideal for inter-molecular interactions with flexible bi-, tri-, and/or tetra-antennary glycans” that terminate in sialic acid. Because the bent-in CD22 molecules have relatively weak interactions within the *cis* nanoclusters, contact with other cells could lead CD22 to redistribute to sites of cell contact and via its elongated rod bind to ligands in *trans*.

## Role of CD22 in response to antigens and pathogenic products

CD22 has been implicated in the regulation of B cell responses to T cell-independent (TI) type 2 antigens (Ags), TLR agonists and T cell-dependent (TD) Ags.

Antibody (Ab) responses to TI-2 Ags are impaired in CD22^−/−^ mice (9–11), perhaps because they are deficient in marginal zone (MZ) B cells and MZ B cell precursors (34, 35). Just why MZ B cells require CD22 is unclear. One possibility is that they are more sensitive to dysregulated signaling in the absence of CD22 (34); but it is also noteworthy that MZ precursors express the highest levels of CD22 of any B cell subset (35), implying that CD22 may be more or less required during stages in B cell development. Mice expressing all of CD22 except the extracellular domains 1 and 2 (CD22Δ1-2 mice) have reduced MZ B cells but normal TI-2 Ab responses (15, 36, 37), so a MZ B cell deficiency alone is not sufficient to lead to impaired TI-2 Ab responses. Recently, Haas et al. (36) reported that B-1b cells from CD22^−/−^ mice have impaired proliferative responses and elevated Ca^2+^ responses to anti-IgM ligation and that CD22^−/−^ mice have reduced expansion of splenic B-1b B cells after immunization with TNP-Ficoll. There results suggest that whether or not CD22^−/−^ mice have defective TI-2 Ab responses depends on the Ag complexity and route of administration used.

Ab responses to LPS are elevated in CD22^−/−^ mice (9–11), and CD22^−/−^ B cells proliferate *in vitro* more strongly than WT B cells to TLR7 (R848) and TLR9 (CpG) agonists (38, 39). Kawasaki et al. (39) showed that CD22^−/−^ B cells also are hyperproliferative to the TLR3 agonist poly I:C and that some of this hyperproliferation, unlike the hyperproliferation to TLR4 and TLR9 agonists, is MyD88-independent. TLR agonists also induced larger increases in MHC class II and CD86 in CD22^−/−^ B cells than WT B cells, suggesting that B cells with dysregulated CD22 may more readily become effective Ag presenting cells, possibly to autoAgs. Kawasaki et al. concluded that this hyperresponsiveness to TLR agonists was not due to CD22^−/−^ B cells expressing higher levels of TLRs; rather their results suggest that CD22 may normally function during TLR signaling of B cells to activate suppressors of cytokine signaling (SOCS) SOCS1 and SOCS3 proteins that are known to blunt responses to TLR ligands. Thus, CD22 normally may play a role in the direct inhibition of TLR signaling in B cells. The natural ligands for CD22 apparently do not play a direct role in regulating proliferative responses to TLR agonists since CD22 ligand-deficient ST6Gal1^−/−^ B cells have normal responses to LPS and CpG (40). CD22 is an endocytic receptor that recycles between the cell surface and the endosomes, where endosomal TLRs resides (41). A model proposed by Paulson et al. (42) suggests that sequestration of CD22 and/or other changes in the CD22 microdomain organization may affect CD22 concentrations in the endosomes and further affect endosomal TLR signaling.

The role for CD22 in TD Ab responses is controversial. Initial studies reported that CD22^−/−^ mice have normal responses to TD Ags (9–11); however, mice were evaluated for short times following immunization, and Ag boosts were administered before primary immune responses had subsided. Ligands for CD22 have been identified on CD22 itself and on T cells (28, 30, 43). Thus, CD22 may engage CD22 ligands in *trans* on T cells and affect T cell activation (14, 44). Furthermore, ST6GalI^−/−^ mice unable to express CD22 ligands have normal T cells but defective TD Ab responses to Ag + adjuvant or influenza infection (12, 45). B cell proliferation induced via the “T cell-help” CD40 receptor is elevated in CD22^−/−^ B cells (37). CD22 also affects intracellular free calcium released by IgG^+^ B cells (46, 47), again implying that CD22-CD22L interactions may influence TD B cell responses.

A recent study suggested that CD22 plays a role in the generation of memory B cells in response to a TD Ag. CD22^−/−^ B1-8^hi^ B cells with a BCR specific for the hapten, 4(hydroxy-3-nitrophenyl)-acetyl (NP) were able to respond to immunization with a TD Ag (NP-CGG in alum) and develop into germinal center (GC) B cells; however, they did not differentiate efficiently into memory B cells or long-lived plasma cells (LLPCs) or sustain Ab levels over time (48). The lack of GC B cell output was associated with a failure of CD22^−/−^ B cells to develop a subset of CXCR4^+^CD38^+^ GC B cells, which may be GC-derived precursors of memory B cells and LLPCs.

In contrast, Onodera et al. (49) reported that after immunization CD22^−/−^ B cells, including GC B cells, rapidly expand and generate short-lived AFCs and antibodies. Unlike in Chappell et al. the recipient mice were previously immunized with CGG (“carrier primed”). Thus, both CGG-specific Tfh cells and CGG-anti-CGG immune complexes that can be taken up by FcγR^+^ cells may have contributed to the rapid hyperproliferative and extrafollicular B cell responses observed, as has been reported (50). Such hyperproliferation was not evident in the bona fide primary immune responses (48). Nevertheless, both studies suggest that CD22^−/−^ B cells do not efficiently generate memory B cells.

SHP-1, which can be recruited to bind CD22, plays a role in GC maintenance and memory cell development (51, 52). Thus, it is possible that the absence of CD22 leads to decreased SHP-1 recruitment required for efficient memory B cell development. Unlike SHP-1 deletion, however, GCs are not completely ablated in the absence of CD22; rather, a small subset of CXCR4^+^CD38^+^ PNA^+^ GC B cells fail to develop that normally appear early in the immune response (48). Cognate interactions between B and T cells are critical for GC initiation and maintenance, and CD22 ligands (CD22Ls) are expressed on T cells as well as B cells (30, 53). Interestingly, a recent study showed that CD22 on naïve and memory B cells is masked through interactions with “high affinity” ligand (Neu5Ac2– 6Gal1– 4(6S)GlcNAc); however, loss of the 6S sulfate modification on GC B-cells results in the appearance of Neu5Ac2– 6Gal1– 4GlcNAc glycans with a lower affinity for CD22 (54). Thus, it is possible that once CD22 is unmasked on GC B cells, CD22L-CD22 interactions may then occur *in trans* between CD22L^+^ CD4 T_FH_ cells and CD22^+^ GC B cells to promote further B cell survival and maturation. CD22^−/−^ GC B cells that are not capable of receiving this type of “help” from T_FH_ cells may not be as competent as WT B cells for memory B cell formation. Thus, in addition to altered BCR signaling, defective interactions between B and T cells may also contribute to the lack of memory formation by CD22^−/−^ B cells.

Hass et al. found CD22^−/−^ mice have elevated IgM and IgG Ab primary and secondary responses to DNP-KLH, while Jellusova et al. found that CD22^−/−^ mice have reduced primary Ab responses to NP-OVA in alum (36, 38). How can these differences be explained? Given that CD22 clearly regulates innate immune and TI signaling, one possibility is that role CD22 plays depends of the nature of the Ag or adjuvant used with the Ag. CD22 may be an attenuator of Ab responses, which does not simply function along a TD vs. TI dichotomy.

## Role of CD22 in migration and other trans interactions

CD22 has long been known to be an adhesion molecule (1). But recent studies from Eugene Butcher's group at Stanford have uncovered a surprising and new role for CD22 in B cell homing to gut associated lymphoid tissues. The Peyer's patches (PP) in the gut are major site for B cell responses to intestinal Ags and attract large numbers of circulating B cells. TheSt6Gal1 ligand for CD22 is selectively expressed on mouse PP high endothelial venules (HEVs) and not on peripheral lymph node (LN) HEVs or on endothelial cells in capillaries (55). Homing to PP is dramatically reduced in both CD22^−/−^ and ST6Gal1^−/−^ mice. An Ab specific for human St6Gal1 binds to mucosal lymphoid organs (56), suggesting that homing to human GALT may be regulated by CD22 as well.

CD22^−/−^ mice are highly susceptible to infection by West Nile virus (WNV) and die within 10 days post-infection (57). Humoral immune responses are normal in WNV-infected CD22^−/−^ mice; however, homing to draining LNs in infected mice is defective. Fewer CD22^−/−^ NK cells, CD4 T cells and CD8 T cells enter LNs than WT counterparts, while migration of CD22^−/−^ B cell and dendritic cells (DCs) is normal. These results suggest that CD22 may regulate cell migration not simply by CD22L-CD22 interactions, but also indirectly, perhaps via regulation of chemokine or chemokine receptor expression. Indeed, the draining LNs of WNV-infected CD22^−/−^ mice had reduced expression of both *Ccl3* and *Ccl5* genes (57).

CD22 also plays a role in the migration of recirculating B cells to the bone marrow (BM). Although B cell development in the BM is not affected in CD22^−/−^ mice, numbers of recirculating B220^hi^sIgM^lo^ B cells (or IgD^hi^ B cells) are reduced in CD22^−/−^ mice (9, 10) as well as CD22L deficient ST6Gal1^−/−^ mice (58). The endothelial cells in BM sinusoids express the α2,6-linked sialic acid ligand for CD22 (59). WT B cells adoptively transferred into ST6Gal1^−/−^ mice have reduced migration to the BM but not to the spleen (58).

DCs can directly regulate and activate B cells (60), and CD22 can bind to ligands expressed on DCs. Immature DCs but not mature DCs can inhibit B cell proliferation in a contact-dependent manner that requires CD22 expression on B cells (35, 61). Immature DCs can also inhibit TLR2- or TLR4-induced proliferation of mouse B cells via a contact and CD22-dependent mechanism (61). Surprisingly, ST6Gal1^−/−^ DCs were just as efficient as wildtype DCs in inhibiting B cell responses to either BCR-ligation or LPS (35, 61), suggesting that CD22 may mediate inhibition of B cells through an interaction not dependent on ST6Gal1. Two groups found that murine CD22 is expressed on a subset of splenic CD8α^−^ DCs (57, 62). CD22 has also been reported to be expressed on human plasmacytoid DC tumors and follicular dendritic cells (63, 64). It is not clear how non-B cell CD22 might function.

## CD22 and infections

Although CD22 regulates multiple B cell functions, the role of CD22 in protection against viral pathogens is unclear. For example, CD22^−/−^ mice infected with lymphocytic choriomeningitis virus, vesicular stomatitis virus (65), or *Staphylococcus aureus* (66) have no differences in survival compared to wild-type (WT) mice. CD22-deficiency not only leads to increased susceptibility to WNV (55), but also accelerates murine AIDS MAIDS induced by a murine leukemia virus (67), CD22^−/−^ mice had a more rapid onset of splenomegaly and lymphadenopathy 4 weeks after infection.

## CD22 and autoimmunity

While B cells are critical for protection against pathogens, they can also contribute to harmful immune responses in many autoimmune diseases by producing Ab directed toward self-Ags, by presenting self-Ags and producing pro-inflammatory cytokines. A number of studies in human and mouse SLE have shown that hyper-responsiveness of B cells due to defects in the regulation of BCR signaling or increased signaling thought the nuclear-sensing TLRs can alter the selection of autoreactive B cells and promote the production of pathogenic auto-Abs (68). CD22 contributes to the regulation of autoimmunity. Some recent data suggest that targeting CD22 can suppress pathogenic B cell responses.

## Cd22 alleles and CD22 deficiency in mice

Several studies in autoimmune-prone mice have identified *Cd22* as a candidate gene associated with susceptibility to lupus-like disease (69, 70). Mapping of autoimmune loci in B6.NZW (New Zealand White) x B6.Yaa (Y-linked autoimmune accelerator) F1 backcross males revealed the presence of a major autoimmune locus on chromosome 7 in the vicinity of *Cd22*^*a*^. This allele was associated with the production of IgG anti-DNA autoantibodies and the development of glomerulonephritis (69).

This brings us back to the original isolation of genomic clones of mouse *Cd22* which demonstrated the presence of at least two (or more) *Cd22* alleles (71). The *Cd22*^*a*^ allele expressed in DBA/2J, DBNl, NZB, and NZC mice has a distinct polypeptide coding sequences, as compared to the *Cd22*^*b*^ allele, expressed in BALB/c, B10, C3H, and C57BL mice (71). This is due to the presence of a restriction fragment length polymorphism (RFLP) within the *Cd22* gene. The two allelic forms of *Cd22* (*Cd22*^*a*^ and *Cd22*^*b*^) differ in the exons encoding the distal extracellular region of mCD22, suggestive of functional differences between the two CD22 isoforms. Others studies confirmed that lupus-prone NZB and NZW mice carry the *Cd22*^*a*^ allele (70, 72) and later, the expression of a third *Cd22* allele, *Cd22*^*c*^, was described in autoimmune prone BXSB mice and the parental SB/Le strain. Similar to the “autoimmune” *Cd22*^*a*^ allele, the *Cd22*^*c*^ showed differences in the distal extracellular regions constituting the ligand-binding domains of CD22. Mary et al. (70) found that, in addition to the wild-type *Cd22* transcripts, *Cd22*^*a*^ and *Cd22*^*c*^*-*alle bearing autoimmune mice express abnormally processed *Cd22* mRNA transcripts; this was due to the presence of interspersed nucleotide element (B1-, B4-, and ID) insertions, a class of retrotransposons, found in intron 2 of the Cd22^a^ and *Cd22*^*c*^ alleles that are not present in the non-autoimmune (*Cd22*^*b*^) allele. Sequence analysis of aberrant *Cd22* mRNA *Cd22*^*a*^ revealed that some of the mRNAs produce truncated forms of CD22 and others might not be expressed at all due the presence of premature stop-codons. These data suggest that the expression of *Cd22*^*a*^ and other alleles can result in lower CD22 expression. The presence of the defective mRNA transcript was further associated with a reduced ability of LPS-activated B cells to up-regulate CD22 (70). Studies by Nitschke et al. (72) showed that CD22 encoded by the *Cd22*^*a*^ allele expressed on B cells in lupus-prone mice is less efficient in binding to CD22L as compared to the *Cd22*^*b*^ counterparts. A significant portion of CD22 in *Cd22*^a^ mice was constitutively unmasked and did not bind surface *cis-* ligands. Similar to Mary et al., the Nitschke group showed that CD22 expression on *Cd22*^*a*^ B cells is lower both in a steady-state condition and upon B cell stimulation. As a result, *Cd22*^*a*^ B cells display a constitutively active phenotype, similar to the phenotype of B cells expressing a mutant CD22 missing its ligand-binding domain (72).

An initial study showed that aged CD22^−/−^ mice have increases in auto-Ab production (73). However, since the CD22^−/−^ mice used were generated using 129/Sv embryonic stem (ES) cells, it is possible that 129-derived loci may have contributed to the autoimmune phenotype (74). Other studies show that CD22^−/−^ mice generated using C57BL/6 ES cells do not develop an autoimmune phenotype spontaneously (9). CD22 deficiency however does accelerate the development of autoimmunity in autoimmune-susceptible mice, Mary at al. showed that crossing CD22^−/−^ mice onto mice carrying the *Yaa* locus, which predisposes mice to develop lupus-like disease due to duplication of *TLR7* and other genes, significantly increased auto-Ab production (70). This study also demonstrated a *Cd22* “gene dosage” effect, since even a partial reduction of CD22 expression (i.e., in heterozygous CD22^+/−^ mice) increased auto-Ab production. Another interesting study showed that deletion of *Cd22* in anti-DNA transgenic (D42HTg) mice rescued autoreactive cells from peripheral toleralization and further promoted the production of high-affinity, class-switched anti-DNA Auto-Abs (75).

The fact that deletion of *Cd22* alone might not be sufficient to drive autoimmune disease in some mice can be explained by some functional redundancy between CD22 and Siglec-G, another Siglec family member, expressed on B cells also implicated in the regulation of BCR signaling (2, 76, 77). Unlike other autoimmune models, *Cd22* deficiency does not promote significant changes in B cell development, except for a decrease in MZ B cells (34). One alternative explanation for the decrease in MZ B cells is that CD22^−/−^ MZ B cells might be partially activated. Similar egress of MZ B cells can be found in other autoimmune models, particularly those associated with TLR7 overexpression (78, 79). The role of CD22 in regulating MZ B cells and a possible link between MZ B cell decrease and increased autoimmunity needs further elucidation.

## CD22 gene variants in human autoimmune diseases

Genetic variants of CD22, or enzymes involved in the glycosylation of ligands of CD22 have been linked to susceptibility in human autoimmune diseases (80, 81). One example is the loss-of-function mutations in the enzyme sialic acid esterase (SIAE), which mediates the deacetylation of N-glycan sialic acids of CD22 ligands, a modification that enables ligand binding to CD22. These rare mutations were found more frequently in patients with autoimmune diseases, such as rheumatoid arthritis (RA), type 1 diabetes (T1D), and SLE (82–84). Furthermore, *Siae* mutant mice display defects in B cell tolerance and spontaneously develop autoantibodies, further supporting the link to autoimmunity (85).

Polymorphisms in the *CD22* gene itself have also been linked to autoimmunity. Hatta et al. (81) performed a systematic variation screening of the human *CD22* gene and studied possible associations between *CD22* polymorphisms and susceptibility to RA and SLE. They identified more than 13 SNPs within the *CD22* locus, the majority of which fell within the coding sequence, and some within introns flanking the exon-intron junctions. Seven of the SNPs resulted in amino acid substitutions within the extracellular domains of CD22. Among these variations, the Q152E substitution was more frequently found in SLE patients, particularly, those with central nervous system (CNS) involvement. Although the association of the Q152E variant with SLE was only marginally significant (81), of interest is that the Q152E substitution is located within the CD22 extracellular domain (at the interface between Ig domains 2 and 3) and introduces a charge difference; since it is located far from the SA-binding pocket, it is unlikely to directly affect CD22 binding to α2-6 sialylated ligands; however, this polymorphism might affect other aspects of CD22 biology such as stability, adhesion and trafficking. Another CD22 polymorphism, identified by Hatta et al. is a non-conservative amino acid substitution (G745D) within the cytoplasmic domain, proximal to a YXXM motif that is a binding site for PI3K (7). While no associations with this polymorphism and SLE or RA disease susceptibility were found, the amino acid change within the CD22 cytoplasmic tail nonetheless might interfere its binding to PI3K, Lyn or SHP-1 and thus, affect CD22 downstream signaling.

A study of patients with cutaneous systemic sclerosis (SSc), an autoimmune disease associated with B cell hyperactivation and the production of autoantibodies, showed a significant association between SSc disease susceptibility and synonymous SNP *c.2304C* > *A (P768P, rs34826052)* located within exon 13 of the *CD22* gene (86). The A/A genotype was present exclusively in patients with limited cutaneous SSc; furthermore, this genotype was associated with a decreased surface expression of CD22 in B cells compared to the A/C and C/C genotypes (86). Studies in a European population, however, found no significant association between *CD22* gene variations, including the *rs34826052* SNP, and susceptibility of SSc (87); this most likely reflects differences in the allele distributions in different populations. In fact data from the 1,000 Genomes project, showed that the *A* allele is found in only 1–3% in Africans, Americans or Europeans, but is more frequent (9–15%) in East Asians and South Asians, which could explain the difference between these studies.

Genome-wide association studies (GWAS) do not support CD22 as disease susceptibility locus in SLE or other autoimmune diseases; however it seems that polymorphisms in *CD*22 are relatively rare and variable between populations. More studies are needed to assess the functional significance of different CD22 SNPs and their possible contribution to autoimmune disease.

## Regulation of CD22 expression with implications in autoimmunity

How the expression of CD22 is regulated in B cells is still not well understood. CD22 on murine B2 cells is down-regulated after BCR cross-linking with anti-IgM mAb, but it is up-regulated after stimulation with other stimuli such as LPS, anti-CD40 mAb, or IL-4. In contrast, BCR crosslinking of CD5^+^ B1 B cells did not change the expression levels of CD22, and B1 cells downregulated CD22 in response to LPS or CpG (88). Thus, CD22 expression is differentially regulated in B1 and B2 cells. CD22 expression can be regulated at the mRNA level (88) or by CD22 endocytosis and recycling. John et al. (89) reported a clathrin-mediated internalization of CD22 and CD22 association with AP50, one of the subunits of the clathrin-associated AP-2 protein adapter complex. Furthermore, BCR crosslinking and CD22 phosphorylation can transiently inhibit CD22 endocytosis. It is not known if upon inflammatory/autoimmune conditions, CD22 mRNA expression and endocytosis is altered. As mentioned above, the presence of *Cd22*^*a*^ allele in mice has been associated with a decrease of CD22 expression.

Relatively few studies have examined the expression of CD22 on B cells from SLE patients; one study reported a decrease in CD22 levels on B cells from SLE patients with active disease; another study showed an association between disease improvement and increased CD22 expression after treatment (90, 91). SSc patients may also have decreases in CD22 expression and reduced CD22 phosphorylation (92). Interestingly, anti-CD22 Abs capable of inhibiting tyrosine phosphorylation of CD22 have been found in both SSc and SLE patients, which might be another, yet-to-be-explored, mechanism for regulation of CD22 function (93).

Another important question is how CD22 expression is regulated during different stages of B cell development and its possible impact on the selection of autoreactive B cells. CD22 is most highly expressed on MZ B cell precursors (35) and remains at high levels on mature B cells; some studies suggest that developing B cells in the BM express low levels of CD22, starting at the Pre-B stage (9). Whereas the numbers of B cell precursors are normal in the BM of CD22-deficient mice, the effects of CD22 on the selection of B cell progenitors have not been studied in detail. Given its role in the regulation of BCR and TLR signaling, it is possible that CD22 may also control the signaling thresholds on developing B cells, and therefore, play a role in the central selection and tolerance induction.

We found that in healthy conditions newly-formed transitional (TR) B cells in both human and mice express relatively high levels of CD22 (Giltiay NV, unpublished data), which might function to prevent unwanted activation, as a number of studies have shown that TR B cells express BCRs that are polyreactive and can bind endogenous antigens (94, 95). Danzer at al. found that the proportion of murine B cells with unmasked CD22 is increased in splenic TR and MZ B cells and peritoneal B1 cells when compared to other mature B cells (96). They proposed that unmasking of CD22 could be functionally involved in lowering the signaling threshold at “developmental checkpoints,” or might be a consequence of cell activation. A combination of predisposing factors such as TLR signals along with the unmasking of CD22 at the TR stage would favor the activation of poly/autoreactive TR B cells and thus contribute to the development of autoimmunity. Of future interest would be to compare the expression (and unmasking) of CD22 on different human B cell subsets in healthy or autoimmune conditions. Studies using auto-Ag-specific BCR transgenic mice lacking *Cd22* might be also useful to study the contribution of CD22 in regulating the selection and activation of autoreactive B cells at different stages of B cell development.

## A role of CD22 in self- and non-self-discrimination

An important question is how CD22-CD22L *cis-* or *trans-* interactions affect the association of CD22 with sIgM and BCR signaling. A model proposed by Cyster and Goodnow (97) suggested that lower levels of sialylated proteins in non-lymphoid tissues promote CD22-sIg associations that “dampen the BCR signaling”; however, when B cells enter a lymphoid environment which is richer in α2,6-sialylated proteins, CD22 might be “drawn away” from sIgM through *trans*-interactions, thus promoting BCR signaling and B cell activation. Such “release” of the BCR from control by CD22 might be necessary when Ag-engaged B cells migrate into the B-cell follicles and interact with T_FH_ cells and form GCs. This model fits well with a study that showed changes in the glycosylation patterns due to altered enzyme activity in the GC, leading to unmasking of CD22 on GC B cells compared naive and memory B-cells, (54).

However, what happens when a B cell binds self-Ag? Some studies proposed that engagement of CD22 may provide a signal to distinguish between self-Ags and non-self, foreign Ags and to prevent self-reactivity (43, 98, 99). It is important to point out that sialyated glycans are abundant in vertebrates' cells/tissues and are usually absent in bacteria. Thus, they can be regarded as “self-structures” (100). Lanoue et al. (98), showed that expression of αST6Gall on self-Ags diminishes the activation of self-Ag-specific B cells, supporting the idea that CD22-2,6-sialoglycoconjugate interactions could bias against B cells being selected by self-Ags arrayed on such cells. More recently, Duong et al. reported that “decorating” TI-2 Ags with native sialylated ligands for CD22 and Siglec-G strongly suppresses antibody responses and promotes a sustained immune tolerance (99). These findings suggest CD22 influences how B cells maintain self-tolerance to cell surface proteins, or secreted high-molecular weight self-Ags.

CD22 also binds soluble self-molecules present in serum. For example, soluble IgM has been proposed as a α2,6-sialylated ligand for CD22 (43). Thus, CD22 might be recruited to the BCR when B cells bind IgM-antigen complexes, or IgM alone and thereby inhibit cell activation by acting as a kind of an inhibitory “IgM-Fc” receptor (97). This might be relevant to therapies using immunomodulatory IVIg. Séïté et al. recently showed that SA-positive IgG, but not SA-negative IgG from IVIg binds to CD22 and can inhibit B cell activation (101).

## A role for CD22 in TLR regulation of B cell responses to auto-ags

Many studies suggest that B cells that recognize self-Ags, especially nuclear Ags, receive second signals from TLRs that recognize DNA or RNA motifs and drive their activation (68). TLR7 and TLR9 in particular have been implicated in the activation of autoreactive cells and antinuclear auto-Ab production in mouse models of lupus (78, 79, 102, 103). Signaling though TLRs can promote multiple functions of B cells, including cytokine production, cell differentiation, class-switch and Ab production. CD22-null mouse B cells have increased proliferative responses to TLR4, TLR7 or TLR9 ligands (38, 39); CD22 as noted above may inhibit TLR signaling in part by reducing the expression of SOCS1 and SOCS3 (39).

We found that engagement of CD22 on human B cells by anti-CD22 Ab inhibits the expression of *PRDM1* in response to TLR7 ligand or a combination of anti-IgM plus TLR7 ligand stimulation (104). *PRDM1* encodes Blimp1, a key transcription factor required for B cells to mature into antibody-secreting plasma cells. CD22 ligation limited B cell differentiation into plasmablasts in response to TLR7 ligation, suggesting that *in vivo* CD22 may function to inhibit TLR7-driven B cell activation of autoreactive B cells. Engagement of CD22 also affects cytokine production by human B cells in response to TLR7 or TLR9 stimulation. CD22 ligation inhibited IL-6 production and increased IL-10 production (104, 105), which might further inhibit pathogenic B cell responses. Mechanistically, CD22 engagement by antibody can induce MAPK/ERK phosphorylation, which can turn on the production of IL-10 (106, 107).

**Figure 1:**
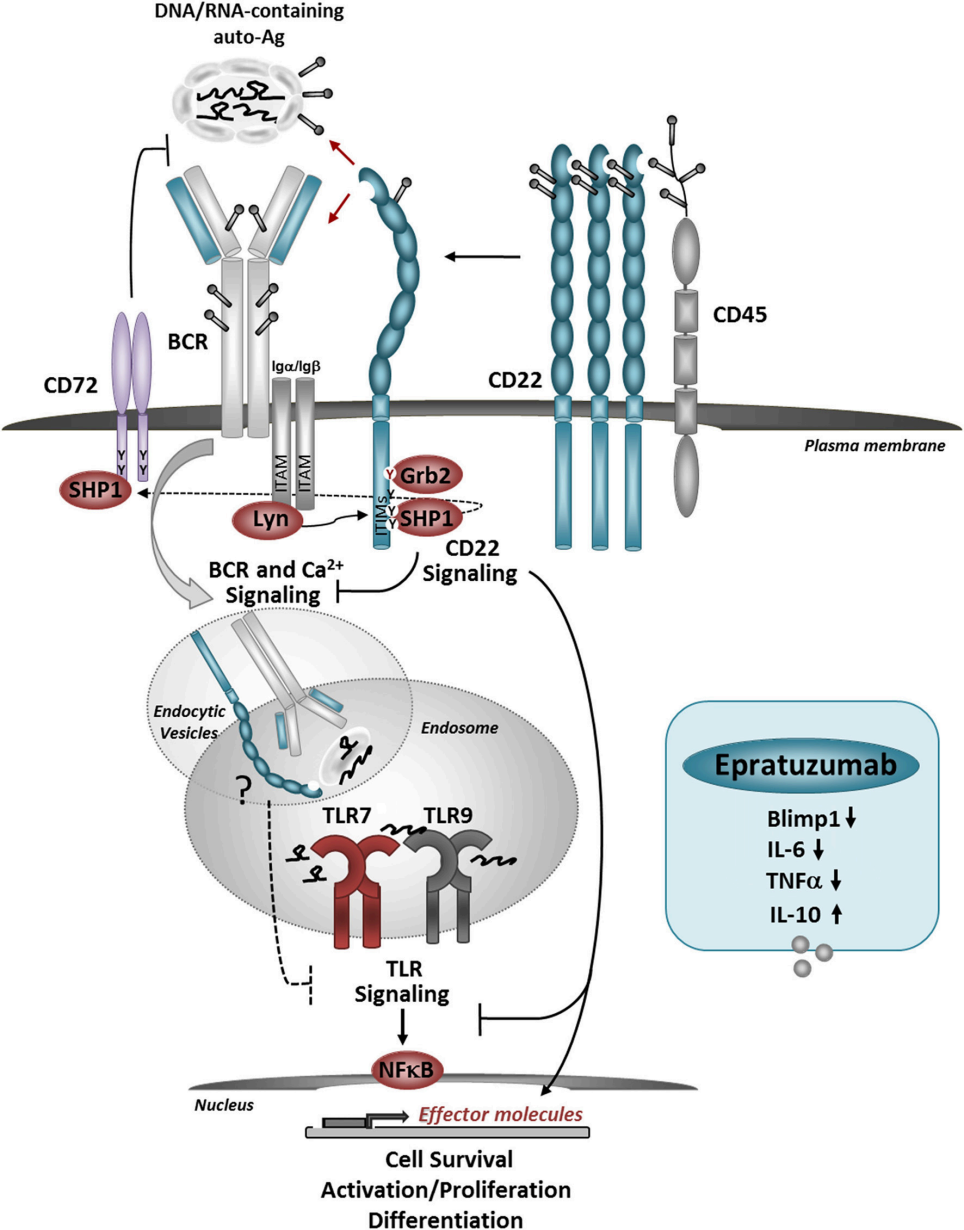
Model for the role of CD22 in regulating BCR/TLR-mediated B cell responses to autoantigens. CD22 molecules are organized in nanodomains, regulated by interactions with CD45. Self-Ags, decorated with sialylated ligands may recruit CD22 molecules close to the BCR, upon which CD22-SHP-1 activation inhibits downstream signaling. The uptake up of nuclear-containing Ags triggers TLR7 and TLR9 activation in the endosomes. CD22 may inhibit TLRs activation *via* several mechanisms: promoting the activation of CD72, direct inhibition of TLR signaling after internalization, and/or affecting the expression and activation of NF-κB and pro-survival pathways. Crosslinking of CD22 with a therapeutic antibody Epratuzumab, inhibits the expression of Blimp1 and pro-inflammatory cytokines in response to TLRs stimulation. Antibody-mediated CD22 ligation induces internalization of CD22, SHP-1, and Grb2 activation and may also promote co-localization with TLRs in the endosomes.

A balance between TLR7 signaling and CD22/CD22L interactions might be important for maintaining self-tolerance and keeping autoreactive B cells “in check” (Figure 1). Since a large proportion of peripheral B cells are poly/autoreactive, and presumably can encounter nuclear Ags in the form of nuclear debris of dying cells, CD22 ligation might be an important mechanism for limiting BCR and TLR signaling. Some studies suggest that cells undergoing apoptosis have a reduced surface expression of α2,3- and α2,6-linked sialic acids, which could affect CD22-*trans* binding and possibly-limit the ability of CD22 to regulate BCR and/or TLR7 responses (108). This might occur in SLE where the accumulation of necrotic/apoptotic cells and improper clearance play a major role in promoting immune cell activation (109). Genetic factors affecting CD22 or TLR7 expression may affect the ability of CD22 to regulate TLR7-mediated B cell activation and contribute to autoimmunity. Inflammatory conditions such as viral infections associated with type I IFN production, increase *TLR7* expression and can promote changes in protein glycosylation, and further affect CD22/TLR7 crosstalk. Increased expression of TLR7 promotes the expansion and activation of newly-formed TR B cells, which, may contribute to the production of anti-RNA/RNP auto-Abs (79, 95). Increased TLR7 signaling and dysregulation of CD22/CD22L interactions may further affect the activation of TR B cells.

It will be important to further explore the mechanisms for CD22-mediated inhibition of TLR7-indiced B cell activation. For example, it is possible that a crosstalk between TLR7 and CD22 might occur after CD22 internalization. Interaction between CD22 and TLR7 might depend on the CD22 “cargo” in the endosomal compartment and may be affected by changes in CD22 microdomain localization. CD22 ligation by self-Ags possibly could add to co-localization with TLR7 in the endosomes and promote inhibition on TLR7 signaling. A recent study showed that CD72 binds to endogenous TLR7 ligand Sm/RNP and inhibits TLR7-driven B cell responses (110). This finding is in line with previous studies which showed that CD72 deficiency in mice causes lupus-like disease, and associations between CD72 polymorphisms with SLE (111). CD72 and CD22 share similar signaling molecules; furthermore, the ITIM motif of CD72 can be a substrate for SHP-1, possibly downstream of CD22. In addition to that, CD22 signaling might interfere with B cell survival and cell-proliferation induced downstream of TLR7 by affecting the activation of NF-κB and pro-survival molecules, such as Bcl_XL_ and Mcl1 (106). Siglec-G/10 has been shown to suppress TLR4 signaling and NFkB activation by forming a complex with CD24, which binds endogenous TLR ligands such as HSP and HMGB1, but not LPS, providing selective repression of the inflammatory responses to Danger-Associated Molecular Patterns (DAMPs), but not pathogen-associated molecular patterns (PAMS) (42, 112).

## CD22: a target for immunotherapy

CD22 is expressed on the surface of most B-cell leukemias and lymphomas and therefore has been explored as a target for Ab-based therapies [reviewed by (113, 114)]. The first fully-humanized anti-CD22 IgG1 antibody, Epratuzumab (Emab) has been evaluated in clinical trials of B-cell NHL and ALL (113). Unlike Rituximab, which depletes circulating B cells, Emab does not induce complement-dependent cytotoxicity or Ab-dependent cellular cytotoxicity (115). While Emab is not very potent in killing malignant B cells as a single agent, positive results were reported when it was used in a combination with Rituximab and different types of chemotherapy (114, 116). Another anti-CD22Ab (HB22.7), which binds the ligand-binding domains of CD22, is under investigation (114). Upon antibody binding, CD22 is internalized, and because of that, it has been utilized for targeting Ab-drug conjugates (ADCs) or immunotoxins. The use of inotuzumab ozogamicin, which combines an anti-CD22 mAb with calicheamicin, an enediyne antibiotic, which, binds DNA and causes DNA breakage is now approved for use in relapsed or refractory B-cell precursor ALL. Another drug, Moxetumomab Pasudotox, which combines anti-CD22 with PE38, a fragment of Pseudomonas exotoxin A, has shown efficacy in patients with hairy cell leukemia (HCL) (114). Other approaches for targeting CD22 in B cell malignancies have utilized high-affinity CD22 ligands (117, 118). Recently, the use of chimeric antigen receptor (CAR) T-cell therapy, specific for CD22 was reported to provide high response rates for patients with B-cell acute lymphoblastic leukemia (B-ALL) who had failed chemotherapy and/or a CD19-targeted CAR T-cell treatment (119).

There has been a significant interest in adopting CD22-targeted agents, such as Emab as therapies for autoimmune diseases, and in particular, for SLE (120, 121). The safety and efficacy of Emab in SLE have been evaluated in several clinical trials (120). Since Emab potentiates reduced BCR signaling and B cell activation, it was predicted to have potent immunomodulatory effects and a good safety profile. Indeed, Phase I and II clinical trials demonstrated clinically relevant, sustained improvements in patients with moderate-to-severe SLE and with no significant side effects (121). However, Emab did not reach its primary clinical endpoint at phase III clinical trial in SLE (122). A very high placebo response and early rescue of non-responders with increased doses of glucocorticoids might have confounded the results of this particular trail. A post-hoc analysis of the Phase III trial showed improved SLE disease activity in response to Emab in a subgroup of SLE patients with associated Sjogren's Syndrome (SjS), suggesting a future use of Emab (123) or other CD22-based drugs.

The mode-of-action of Emab in SLE is not fully understood. CD22 ligation by Emab induces rapid internalization and phosphorylation of CD22, inhibition of Syk and PLCγ2, and reduces intracellular Ca^2+^ mobilization after BCR stimulation in *vitro* (124). Emab-induced CD22 phosphorylation also enhances its co-localization with SHP-1 and Grb2 (125). Epratuzumab induces a partial reduction of circulating B cells in SLE patients, which might be associated with the effects of Emab on the expression of the adhesion molecules such as CD62L, β7 integrin, and β1 integrin and changes of B-cell migration (124).

Emab affects the production of cytokines in response to BCR/TLR stimulation, by skewing B cells to produce immunoregulatory cytokines such as IL-10 while inhibiting IL-6 and TNF alpha production (104, 105). Thus, targeting CD22 may restore IL-10 production by regulatory B cells, reported to be impaired in SLE patients (126). Emab inhibits the activation and the expression of PRDM1/Blimp1 in response to BCR and TLR7 stimulation in a subset of CD27^−^IgD^−^ double-negative (DN) memory B-cells (104), known to be elevated in SLE patients with more active disease (127).

More work needs to be done to understand the possible therapeutic effects of CD22-based drugs in SLE and potentially to predict which patients respond to CD22-mediated therapies; genetic factors, including defects in CD22 and CD22L, might play a role in responsiveness to CD22 targeting. Recently, Ereño-Orbea et al. delineated the CD22 site targeted by Emab and showed that glycosylation of CD22, which might be altered in B-cell malignancies and autoimmune conditions such as SLE, can affect the ability of Emab to bind its epitope on CD22 (33).

Macauley et al. (128) used liposomal nanoparticles bearing a synthetic high-affinity ligand for CD22, which contained optimized ratios of Ag that can deliver Ag to B cell while engaging CD22. The administration of these SIGLEC-engaging Ag-liposomes (STALs) in mice decreased Ab responses upon a second challenge with the same particles without CD22L, suggesting the induction of Ag-specific immunogenic tolerance (128, 129). The authors showed that STAL-induced B cell tolerance was associated with CD22-mediated inhibition of BCR signaling and recruitment of SHP-1. The potential of STALs was also demonstrated in a mouse model of hemophilia A, which showed a sustained inhibition of anti-VIII Ab responses after mice were administered recombinant FVIII replacement therapy. Another study in MRL/lpr mice has demonstrated the use of chimeric antibody constructed by coupling copies of a DNA mimotope peptide and CD22-binding STN peptide to a mouse IgG backbone. This triple chimera targeted selectively autoreactive B cells and the simultaneous engagement of the BCR, CD22 and, FcgRIIb inhibited anti-DNA Ab production and delayed the development of disease (130). Targeting anti-CD22 was also shown to partly deplete and reprogram B-cells in autoimmune NOD mice, thereby reversing the development of autoimmune diabetes (131). Recently STALS targeting hCD22 ligand were reported to induce to immunological tolerance in humanized CD22 Tg mice. This new model may provide a valuable tool to study the function of human CD22 *in vivo* and for future preclinical studies (132).

## Conclusions

CD22 plays a key role in affecting B cell responses to Ags and innate immune signals, and CD22-CD22L interactions are essential for maintaining self-tolerance. Despite the evidence implicating CD22 in murine lupus, human genetic studies do not support *CD22* as a major disease susceptibility locus in SLE. However, it is likely that defects in *CD22* combined with other genetic factors have additive or synergistic effects on disease susceptibility. The ability of CD22 to regulate both BCR and TLRs represents an attractive therapeutic strategy for manipulating B cell responses in autoimmunity. A challenge for the future would be to fully understand the mode-of action of different CD22-tarageting agents. New methods for CD22-mediated targeting of pathogenic autoreactive B cells without compromising the host's ability to respond to foreign pathogens are a potential new exciting avenue for immunotherapies.

## Author contributions

EC wrote sections of the review and edited sections written by NG, completed final manuscript. NG wrote section of the review and edited sections written by EC, prepared Figure.

### Conflict of interest statement

The authors declare that the research was conducted in the absence of any commercial or financial relationships that could be construed as a potential conflict of interest.
